# Human fetal globin gene expression is regulated by LYAR

**DOI:** 10.1093/nar/gku718

**Published:** 2014-08-04

**Authors:** Junyi Ju, Ying Wang, Ronghua Liu, Yichong Zhang, Zhen Xu, Yadong Wang, Yupeng Wu, Ming Liu, Loretta Cerruti, Fengwei Zou, Chi Ma, Ming Fang, Renxiang Tan, Stephen M. Jane, Quan Zhao

**Affiliations:** 1 The State Key Laboratory of Pharmaceutical Biotechnology, School of Life Sciences, Nanjing University, Nanjing 210093, China; 2 Department of Medicine, Monash University Central Clinical School, Prahran, VIC 3181, Australia; 3 Department of Chemistry, Northwestern University, Evanston, IL 60208, USA; 4 Institute of Life Sciences, Southeast University, Nanjing 210096, China

## Abstract

Human globin gene expression during development is modulated by transcription factors in a stage-dependent manner. However, the mechanisms controlling the process are still largely unknown. In this study, we found that a nuclear protein, LYAR (human homologue of mouse Ly-1 antibody reactive clone) directly interacted with the methyltransferase PRMT5 which triggers the histone H4 Arg3 symmetric dimethylation (H4R3me2s) mark. We found that PRMT5 binding on the proximal γ-promoter was LYAR-dependent. The LYAR DNA-binding motif (GGTTAT) was identified by performing CASTing (cyclic amplification and selection of targets) experiments. Results of EMSA and ChIP assays confirmed that LYAR bound to a DNA region corresponding to the 5′-untranslated region of the γ-globin gene. We also found that LYAR repressed human fetal globin gene expression in both K562 cells and primary human adult erythroid progenitor cells. Thus, these data indicate that LYAR acts as a novel transcription factor that binds the γ-globin gene, and is essential for silencing the γ-globin gene.

## INTRODUCTION

The human β-globin locus is composed of five globin genes (ϵ-, ^G^γ-,^A^γ- δ-, β-globin) located on the short arm of chromosome 11. Globin genes are expressed in a developmental- and tissue-specific manner. The γ-globin genes (^G^γ,^A^γ) are expressed throughout most of fetal life, and their expression is gradually replaced by β-globin after birth (1). Mutations in the β-globin gene can cause β-thalassemia and sickle cell disease (SCD) (2). Reactivation of γ-globin gene expression in adulthood has proven to be one of the best strategies to ameliorate the symptoms in these patients. Because of the clinical significance of β-globins, numerous studies have focused on the molecular events regulating their expression and the γ to β switch (3,4).

During development, expression of β-globin genes is coordinately regulated by cis and trans acting elements, including DNase I hypersensitive sites (located 6–20 kb upstream of the ϵ-globin gene), DNA binding sites within the promoter of each globin gene and lineage-specific transcription factors or cofactors (3,4). It has been found that transcription factors, such as GATA1, KLF1 (EKLF), NF-E2 and SCL, participate in the developmental modulation of each globin gene and the subsequent erythroid differentiation or cell commitment (5). The transcription factor GATA1 was initially isolated based on its binding to the β-globin promoter, and has since been found to bind to most known erythroid genes (6). GATA1 has two conserved zinc finger domains enabling it to bind the (A/T)GATA(A/G) consensus DNA motif. GATA1 plays an important role in the process of erythroid cell commitment (7,8). GATA1 knock-out mice die from severe anemia at E10.5-E11.5 (9). Another key regulator of erythroid cells is KLF1, which binds to a CACCC box motif (10). Mutations in CACCC boxes in the human β-globin gene correlate with the incidence of thalassemia (11). KLF1 knock-out mice die from anemia at E14-E15 (12,13).

Among all human globin genes, γ-globin genes are unique. Within the γ-promoter, there are a single CACCC box, two CAAT boxes and a canonical TATA box. Before their *in vivo* activities were confirmed, the transcription factors FKLF and FKLF2 were found to bind to a CACCC site at position −145 of the γ-promoter (14,15). The ubiquitously expressed transcription factors CP1 and C/EBP bind to the CAAT boxes at positions −115 and −85, and compete with the repressive protein CDP (CAAT displacement protein) (16). A DR1 (direct repeat) motif adjacent to these sites is bound by the direct repeat erythroid-definitive repressor complex containing TR2 and TR4 (two nuclear orphan receptors), although enforced expression of TR2/TR4 paradoxically enhances fetal hemoglobin synthesis in both murine adult erythroid (AE) cells and SCD model mice (17,18). At position +9, the transcription factors Stat3 and GATA1 act cooperatively to repress γ-globin gene expression (19). Recently, in genome-wide association studies, the transcription factor BCL11A, which regulates HbF levels, was found to bind a G-rich region (GGCCGG) and to suppress γ-globin gene expression (20,21). This finding was supported both by *in vitro* erythroid cell experiments and *in vivo* mouse data (22). Interestingly, KLF1 also regulates γ-globin gene expression indirectly through regulating BCL11A (23).

In previous studies, we showed that a stage selective element (SSE) occurs within positions −53 to −34 of the γ-globin promoter. The SSE can be bound by a stage selective protein complex containing a ubiquitously expressed transcription factor CP2 and an erythroid-specific factor NF-E4 (24–26). Recently, a chromatin regulating complex that includes arginine methyltransferase PRMT5, lysine methyltransferase SUV4–20h1, NuRD complex components and DNA methyltransferase DNMT3A has been shown to coordinately regulate γ-globin gene expression (27,28). The histone mark H4R3me2s, which is triggered by PRMT5, appears to be an early step in silencing γ-globin gene expression. In the current study, we found that PRMT5 interacted with a nuclear zinc finger protein, LYAR (Ly-1 antibody reactive clone) which was first identified two decades ago (29). The results demonstrated that LYAR directly binds the γ-globin gene region corresponding to 5′ untranslated region (UTR), and this binding silences γ-globin gene expression in both K562 cells and human erythroid progenitor cells.

## MATERIALS AND METHODS

### Cell cultures

CD34+ cells were isolated from healthy human adult peripheral blood and cord blood (CB) mononuclear cells by magnetic sorting. CB and adult CD34+ cells were cultured as previously described (20,28). Briefly, isolated CD34+ cells were grown in StemSpan SFEM medium with 1× CC100 cytokine mix for 6 days, and then reseeded into the same medium supplemented with SCF (20 ng/ml), EPO (1 U/ml), IL-3 (5 ng/ml), dexamethasone (2 μM) and β-estradiol (1 μM) and cultured 12 days. On days 6 and 7 of culture, erythroid progenitors from CB were infected with MSCV-HA-LYAR-IRES-GFP retroviral supernatant described below. Infected cells were selected for green fluorescent protein (GFP) expression by flow cytometry on day 9 and cultured 3 days in the same medium prior to harvest. For lentivirus infection of AE cells, progenitors were infected with viral supernatants on days 6, 7 and 8. Transduced cells were selected for GFP expression by fluorescence-activated cell sorting on day 12 and cultured another 6 days. Cell surface marker analysis with CD71 and Glycophorin A (GPA) indicated that more than 90% of cultured cells were of erythroid lineage. Human peripheral blood and CB collection were approved by Melbourne Health and Royal Women's Hospital Human Research Ethics Committees. K562 cells were maintained in RPMI (Roswell Park Memorial Institute-1640) with 10% fetal calf serum (FCS); MEL cells and 293T cells were maintained in Dulbecco's modified Eagle's medium with 10% FCS.

### Cell growth assay and luciferase reporter assay

Cells were seeded at 4000 cells per well (100 μl) in triplicate in 96-well plates. Cell Counting Kit-8 (CCK-8, Dojindo, Japan) solution (10 μl) was added to each well of the plate, and plates were incubated at 37°C, 5% CO_2_, 1 h. Absorbance at 450 nm was measured using a microplate reader. Luciferase reporter assays were performed as described previously (30).

### Mass spectrometry, protein interaction studies and hemoglobin analysis

FLAG antibody immunoprecipitates from PRMT5-FLAG overexpressing K562 cells were eluted with 3× FLAG peptide, separated on sodium dodecyl sulphate-polyacrylamide gel electrophoresis (SDS-PAGE) gels, and stained with SimplyBlue Safestain (Invitrogen). Protein bands of interest were excised and subjected to electrospray–ion trap tandem mass spectrometry (LCQ-Deca, Finnigan). Immunoprecipitation, immunoblotting and Glutathione S-transferase (GST) pulldown assays were performed as described previously (27). Anti-FLAG M2 affinity gel and 3× FLAG peptide were purchased from Sigma. Hemoglobin analysis was performed by using the 2-min alkali denaturation method (31,32).

### Generation of anti-LYAR antibody and immunofluorescence staining assays

Human LYAR cDNA was cloned into pGEX6p-1 vector, and expression of full-length protein was induced in BL21 (DE3) *Escherichia coli* by Isopropyl-β-D-thiogalactopyranoside (IPTG). The GST-tag was removed by treatment with PreScission protease (GE Healthcare Life Sciences). The resulting LYAR protein was purified and used to immunize rabbits to generate anti-LYAR antibody from which the corresponding immunoglobulin G (IgG) fraction was purified (Supplementary Figure S1).

Immunofluorescence analyses were performed as described previously (27). Polyclonal anti-LYAR or monoclonal anti-PRMT5 antibodies were used in the assay.

### CASTing assay

Oligonucleotides R76 comprising a random 26bp core flanked by polymerase chain reaction (PCR) primer sequences were synthesized and used in CASTing experiments: R76, CAGGTCAGTTCAGCGGATCCTGTCG-(N)26-GAGGCGAATTCAGTGCAACTGCAGC; forward primer, GCTGCAGTTGCACTGAATTCGCCTC; reverse primer, CAGGTCAGTTCAGCGGATCCTGTCG. Double-stranded R76 oligonucleotides were prepared by mixing 0.5 μg R76 oligo, 0.3 μg forward primer, 1 μl 10× Klenow buffer, 1 μl 2 mM dNTPs and 1 μl Klenow enzyme (final volume, 10 μl) and incubating at 37°C for 1 h. Binding reactions were performed on 200 μg K562 cell extract with 50 ng double-stranded R76 oligo in CASTing binding buffer containing 20 μg poly(dI-dC) and incubation on a roller platform for 30 min at 4°C. Then, 5–10 μl antibody was added, and incubation was continued for 30 min at 4°C. Protein A-sepharose (50 μl) was then added to the mixture, and following a further 30 min incubation, DNA-protein-antibody complex was pulled down by centrifugation. Pellets were washed four times with binding buffer and then incubated in 200 μl elution buffer at 50°C for 1 h. DNA was recovered from the supernatant by phenol/chloroform extraction and ethanol precipitation, and added to 100 μl PCR reaction containing 10 μl 10× PCR buffer, 10 μl 2 mM dNTPs, 2 μl each forward primer and reverse primer (50 μM each), 1 μl Taq polymerase. The PCR reaction was aliquoted into four tubes for PCR amplification. A reaction tube was removed after 10, 13, 16 and 20 cycles. Samples were analyzed by agarose gel electrophoresis, and the sample in which DNA was first faintly detected were used for the next round of CASTing. After four cycles of CASTing, PCR products were cloned into TOPO-vector, and 69 colonies were chosen for sequencing.

### Electrophoretic mobility shift assays (EMSA)

EMSA was performed using LightShift EMSA optimization and control kit (Pierce, Rockford, IL, USA). Nuclear extracts prepared from K562 cells were incubated in binding buffer for 15–20 min at room temperature with double-stranded oligonucleotides 5′-biotin-AACGTCTGAGGTTATCAATAAGCT-3′ and its complementary strand, which correspond to the sequence on the γ-globin 5′UTR. The reaction samples were run on 6% native polyacrylamide gels in 0.5× TBE (Tris/Boric acid/EDTA buffer) buffer. The binding reactions were transferred to nylon membranes using a Bio-Rad semi-dry transfer apparatus, and DNA was cross-linked to membrane with a ultraviolet illuminator. The biotin-labeled DNA complexes were visualized using a Pierce Chemiluminescent Nucleic Acid Detection Module.

### Lentiviral or retroviral infection

The siRNA target sequences of RNA interference for LYAR were inserted into the *Xho*I/*Hpa*I sites in the pLL3.7 lentiviral vector according to the manufacturer's recommendations (American Type Culture Collection, USA). The oligonucleotides were:

Human LYAR shRNA1: GGTCATCTTTAACAAGAAA
Human LYAR shRNA2: CCTGGTCATCTTTAACAAG
Mouse LYAR shRNA1: CGAGATATCAGTCAAGAAG
Mouse LYAR shRNA2: CAGAGATGCCGATCACTAA

For overexpressing LYAR, human LYAR cDNA was cloned into the retroviral vector plasmid MSCV-HA-IRES-GFP at unique *Xho*I and *BamH*I sites. Lentivirus or retrovirus production in 293T cells and infection of K562 cells or human erythroid progenitor cells were performed as described previously (27,28). Lentiviral infection of MEL cells was similar to that of K562 cells. Transduced cells were selected for GFP expression by flow cytometry.

### ChIP assay

Chromatin Immunoprecipitation (ChIP) assays were performed with K562 cells as described previously (28). Antibodies against histone H3K9ac and H4R3me2s were purchased from Abcam. PRMT5 antibodies were purchased from Sigma. Normal rabbit IgG served as the control. Precipitated DNA was analyzed by Q-PCR. ChIP samples were analyzed by quantitative real-time PCR using the FastStart Universal SYBR Green Master (Roche). A standard curve was prepared for each set of primers using serial titration of the input DNA. The percentage of ChIP DNA was calculated relative to the input DNA from primer-specific standard curves using the Rotor-Gene 6000 Series Software 1.7. ChIP primers are listed in Supplementary Figure S2. Student's *t*-test was used to derive the significance of the differences between mean values.

### RNA isolation and real-time PCR

RNA was isolated from cells with Trizol reagent (Life Technologies) according to the manufacturer's protocol. cDNA was synthesized with the SuperScript first-strand synthesis system (Life Technologies). Real-time quantitative reverse transcriptase (RT)-PCR was performed using the FastStart Universal SYBR Green Master (Roche) in a Rotor-Gene 6000 (Corbett Research) in a final volume of 20 μl. Cycling conditions were 94°C for 15 s, 60°C for 30 s and 72°C for 30 s. Each reaction was performed in triplicate. Primers are listed in Supplementary Figure S2. Student's *t*-test was used to derive the significance of the differences between mean values.

## RESULTS

### LYAR interacts with PRMT5

**Figure 1.**
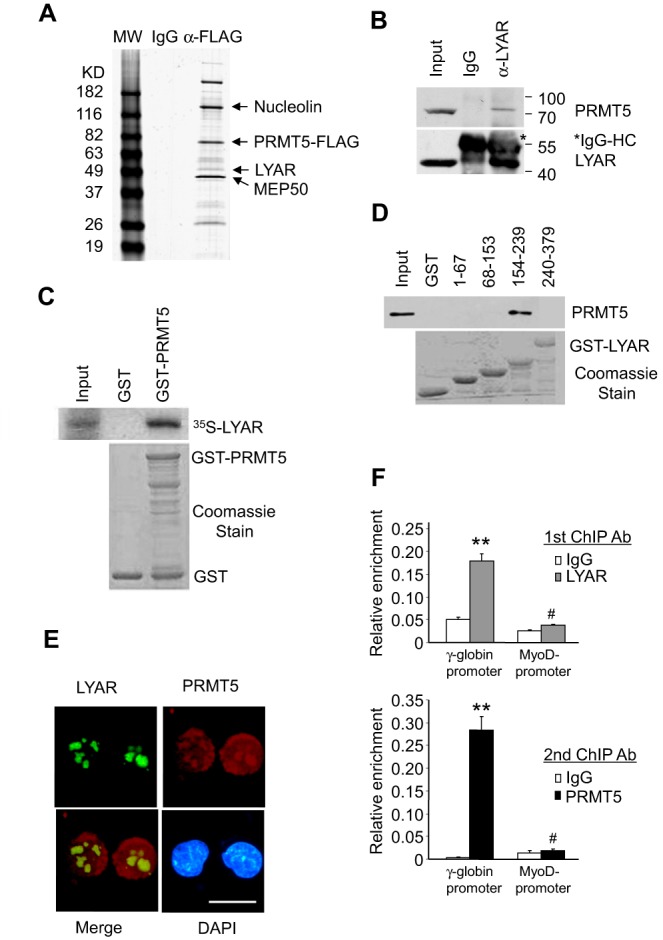
LYAR interacts with PRMT5. (**A**) SimplyBlue Safestain staining of an SDS-PAGE gel of 3x FLAG eluates of anti-Flag antibody immunoprecipitates from K562 cells stably overexpressing PRMT5-Flag. Bands corresponding to Nucleolin, PRMT5, LYAR and MEP50 are indicated. (**B**) Co-immunoprecipitation of endogenous PRMT5 and LYAR from K562 cells. Asterisk indicates immunoglobulin heavy chains. (**C**) GST pull-down assay. Purified GST and GST-PRMT5 fusion proteins (bottom panel, Coomassie stain) pre-adsorbed to glutathione-sepharose beads were incubated with ^35^S-labeled *in vitro* transcribed and translated LYAR. Specifically bound protein was eluted from washed beads and visualized by autoradiography (top panel) after SDS-PAGE. (**D**) Purified GST and GST fusion proteins containing amino acids 1–67, 68–153, 154–239 and 240–379 of LYAR (bottom panel, Coomassie stain) pre-adsorbed to glutathione-sepharose beads were incubated with PRMT5-overexpressing K562 cell lysate. Specifically bound protein was visualized by western blot using anti-PRMT5 antibody after SDS-PAGE. (**E**) Cellular localization of LYAR and PRMT5 in K562 cells was shown by immunofluorescence with anti-LYAR, anti-PRMT5 antibodies and DAPI nuclear counterstaining. Scale bar, 50 μM. (**F**) ChIP-reChIP (anti-LYAR antibody ChIP followed by anti-PRMT5 antibody ChIP) analysis of LYAR and PRMT5 on the γ-globin promoter or MyoD promoter. Normal rabbit IgG served as the control. Data show mean ± SD from three independent experiments. ^#^*P* > 0.05, **P< 0.01 compared to the IgG control.

In previous studies, we have shown that PRMT5 interacts with DNMT3A, thereby coupling DNA methylation and histone arginine methylation on the human γ-promoter to silence γ-globin gene expression (27). To identify other potential proteins interacting with PRMT5, we performed immunoprecipitation experiments using anti-Flag antibody conjugated to sepharose beads to precipitate PRMT5 and associated proteins from cellular extracts of K562 cells stably overexpressing Flag-tagged PRMT5. Proteins were eluted with 3× Flag peptide and subjected to mass spectrometry analysis. We found that, in addition to previously identified PRMT5-interaction partner proteins, such as MEP50 and Nucleolin (33,34), peptides corresponding to a new protein, LYAR (human homologue of mouse Ly-1 antibody reactive clone) were also present (Figure 1A) (29). To confirm the interaction between LYAR and PRMT5, endogenous proteins from K562 cells were analyzed by immunoprecipitation and western blot. We found that PRMT5 was co-immunoprecipitated with LYAR protein from K562 cellular extract by anti-LYAR antibody (Figure 1B). Furthermore, GST pull-down assays demonstrated that these proteins interacted directly (Figure 1C), and their interaction was mediated by amino acids 154–239 of LYAR containing a stretch of mostly charged residues (Figure 1D and boxed sequence in Supplementary Figure S3). Immunofluorescence staining demonstrated that LYAR co-localized with PRMT5 in the nucleus (Figure 1E). As PRMT5 was previously demonstrated to bind the γ-globin promoter, we determined if LYAR also bound the γ-globin promoter. ChIP-reChIP experiments showed that LYAR and PRMT5 together bound the γ globin promoter, but did not bind the MyoD promoter (Figure 1F). Furthermore, enriched binding of LYAR occurred around the proximal promoter and the first exon region of the γ-globin gene (Supplementary Figure S4).

### Identification of LYAR consensus DNA-binding sequences

**Figure 2.**
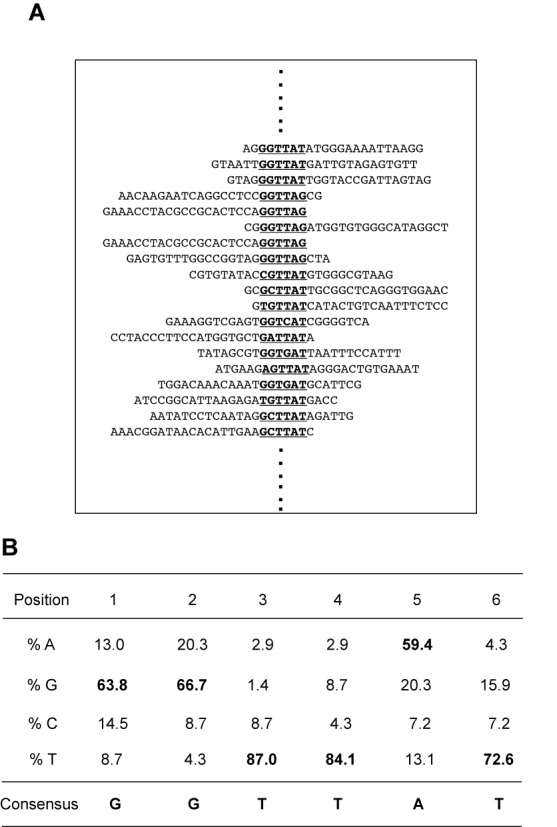
Identification of the LYAR binding consensus sequence. (**A**) Partial DNA sequence alignment of DNA sequencing results from CASTing experiment. (**B**) Binding site preference for LYAR. The frequency of each nucleotide at each position is indicated as a percentage. The putative consensus sequence is 5′-GGTTAT-3′.

LYAR has been shown to have zinc finger DNA-binding motifs (Supplementary Figure S3) (29). Since LYAR was able to bind the γ-globin promoter as demonstrated by ChIP assays, we asked if LYAR was able to bind DNA directly. To test this possibility, we selected LYAR-binding sequences using the CASTing (cyclic amplification and selection of targets) method (35,36). For this purpose, exogenously expressed HA-tagged LYAR from nuclei of K562 cells (Supplementary Figure S1B) was incubated with a pool of double-stranded oligonucleotides containing 26 nt of random core sequences flanked by primer sequences. DNA–protein complexes were immunoprecipitated with anti-HA antibody; the DNA was subsequently recovered, and subjected to 15–20 cycles of PCR amplification. The PCR products were then used in another round of selection and immunoprecipitation. After four such rounds of selection, the final PCR products were cloned, and 69 different clones were sequenced. The sequences obtained were then analyzed by aligning them as shown in Figure 2A and Supplementary Figure S5. We found that LYAR preferentially bound to a consensus motif containing GGTTAT (Figure 2B). Interestingly, the same nucleotide sequence occurs from position +26 to +32 on the γ-globin proximal promoter relative to the CAP site.

**Figure 3.**
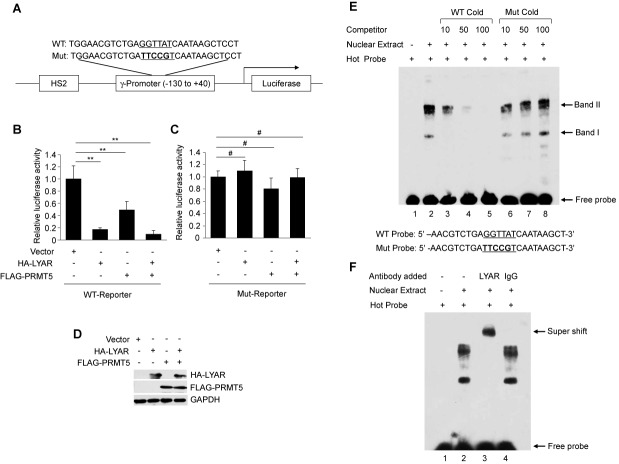
LYAR binds the 5′UTR of the γ-globin gene. (**A**) Schematic representation of a luciferase reporter construct containing either WT or mutant LYAR-binding site. HS2, DNase hypersensitive site 2. (**B**) Relative luciferase activity assay from 293T cells containing either vector, overexpressed HA-tagged LYAR, FLAG-tagged PRMT5 or both. Histograms show mean ± SD, *n* = 3. (**C**) Relative luciferase activity assay as in (B) with mutant promoter. Histograms show mean ± SD, *n* = 3. **P< 0.01, ^#^*P*> 0.05 compared to the empty vector. (**D**) Western blot analysis of HA-tagged LYAR and FLAG-tagged PRMT5 from cell lysates of transfected 293T cells. GADPH was used as a loading control. (**E**) EMSA competition analysis with the indicated amounts (10-, 50- or 100-fold molar excess) of WT cold probe or mutant (Mut) cold probe competitors of K562 nuclear extract. DNA binding bands are indicated by arrows. Sequences of probes are shown below. (**F**) EMSA of K562 nuclear extract using anti-LYAR antibody or control rabbit IgG. Super shifted band is indicated.

In order to test whether LYAR together with PRMT5 functions to transcriptionally regulate gene activity by binding to GGTTAT, we constructed a luciferase reporter gene driven by DNase hypersensitive site 2 (HS2) of the β-globin locus and a minimal γ-promoter (nucleotide −130 to +40) with either wild-type (WT) or a mutant LYAR-binding site (Figure 3A). When the WT reporter was co-transfected with expression vector containing HA-tagged LYAR or FLAG-tagged PRMT5 or both into 293T cells, a significant decrease in relative luciferase activity was observed compared to the empty vector (Figure 3B). However, no significant change was found when the mutant LYAR-binding site reporter was used (Figure 3C). The expression of the exogenous protein was verified by western blot analysis (Figure 3D). This result demonstrated that LYAR could regulate transcription through binding GGTTAT.

To determine if LYAR bound the proximal γ-promoter containing this core element, we performed EMSA using a 24 bp labeled oligonucleotide corresponding to the γ-globin 5′UTR +17 to +40 which included the GGTTAT motif in the +26 to +32 region. Nuclear extracts from K562 cells were prepared and incubated with this probe (Figure 3E, WT probe). We observed two potential LYAR-binding bands (Figure 3E, lane 2, bands I and II). The faster migrating band I could be easily competed with cold WT probe (Figure 3E, lanes 3–5). However, neither band I nor band II could be competed with cold mutant (Mut) probe, even at a 100-fold excess (Figure 3E, lanes 6–8). These results suggested that bands I and II may be LYAR-binding core element-dependent. The slower migrating band II may represent a complex containing LYAR. To verify that the bands contained LYAR bound to the probe, anti-LYAR antibody was used in the EMSA assay. Bands I and II were both supershifted by the antibody (Figure 3F, lane 3), whereas control IgG did not alter migration of either band (Figure 3F, lane 4). These results indicated that LYAR or a LYAR-containing complex bound directly to a DNA sequence containing the GGTTAT motif.

### LYAR represses γ−globin gene expression in K562 cells

**Figure 4.**
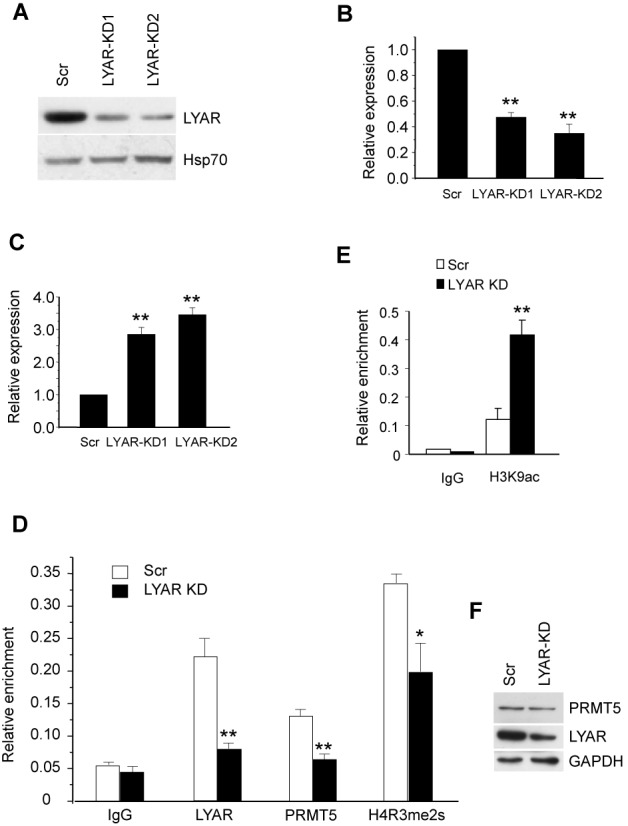
LYAR represses γ-globin gene expression in K562 cells. (**A**) Western blot analysis with indicated antibodies of cell lysate from LYAR-knockdown 1 (KD1) and LYAR-knockdown 2 (KD2) or scrambled (Scr) control K562 cells. (**B**) LYAR gene expression analysis by Q-RT-PCR of RNA extracted from LYAR-KD1, LYAR-KD2 and scrambled control (Scr) K562 cells normalized to β-actin mRNA. Results are shown as mean ± SD from three independent experiments. (**C**) γ-globin gene expression analysis by Q-RT-PCR of RNA extracted from LYAR-KD1, LYAR-KD2 and scrambled control (Scr) K562 cells normalized to β-actin mRNA. Results are shown as mean ± SD from three independent experiments. (**D**) LYAR, PRMT5 and histone H4R3me2s ChIP analyses at the γ-globin promoter were performed in Scr or LYAR-knockdown (KD) K562 cells. Results are shown as mean ± SD from three independent experiments. (**E**) Histone H3K9ac ChIP analysis as in D. *P< 0.05, **P< 0.01 compared to the scrambled control. (**F**) Western blot analysis of LYAR and PRMT5 from cell lysates of LYAR-KD and Scramble K562 cells.

To determine the role of LYAR in γ-globin gene regulation, we generated two stable LYAR knockdown K562 cell lines using lentiviral vectors containing specific shRNAs. LYAR protein levels were assessed by western blot (Figure 4A), and γ-globin gene expression was quantified by Q-RT-PCR from total RNA in these cells. The cell growth rate was not changed when LYAR was knocked down in K562 cells (Supplementary Figure S6). As assessed by Q-RT-PCR, LYAR expression levels were reduced in these two cell lines to about 40% of the Scramble control (Figure 4B), and γ-globin expression was significantly increased compared to the scramble (Figure 4C). Of note, other markers of erythroid differentiation of K562 cells, such as CD71 and v-myb, were not increased in LYAR knockdown cells (Supplementary Figure S9A). To test whether PRMT5 binding to the γ-globin promoter was associated with LYAR binding on the γ-globin promoter, we performed ChIP experiments in LYAR knock-down cells. We found that the levels of PRMT5 binding on the promoter were significantly reduced when LYAR protein was reduced (Figure 4D). The histone mark H4R3me2s, which is induced by PRMT5, was consistently decreased in LYAR knockdown cells (Figure 4D). We also observed an increased level of histone mark H3K9 acetylation on the γ-globin promoter in LYAR knockdown cells (Figure 4E). Of note, the level of PRMT5 was not affected in LYAR knockdown cells compared to the Scramble control (Figure 4F). These data indicate that LYAR repressed expression of human γ-globin, and contributed to PRMT5 binding to the γ-globin promoter in K562 cells.

### LYAR silences mouse embryonic globin gene expression in MEL cells

To test if repression of globin gene expression by LYAR is preserved during mammalian evolution, we constructed lentivirus-based shRNA to knock down murine LYAR in MEL cells, in which embryonic ϵy-globin and βh1-globin are expressed at low levels. Following infection of MEL cells with LYAR shRNA lentivirus and subsequent flow cytometric sorting of GFP positive cells, a significant reduction of LYAR protein was detected by western blot (Supplementary Figure S7A). Q-RT-PCR analysis also confirmed that the LYAR mRNA levels were decreased by shRNA against LYAR (Supplementary Figure S7B). The consequences of LYAR knockdown on murine embryonic globin were examined by Q-RT-PCR. As shown in Supplementary Figure S7B, ϵy-globin was increased significantly in MEL cells following LYAR knock down. βh1-globin was increased to an even greater extent, 5- to 8-fold, in LYAR knockdown MEL cells. The expression of βmajor-globin or βminor-globin gene expression was mildly increased by LYAR knockdown. These results indicate that the presence of LYAR also silenced murine embryonic globin expression in MEL cells.

### Human LYAR protein levels correlate with globin gene expression during erythroid cell differentiation

**Figure 5.**
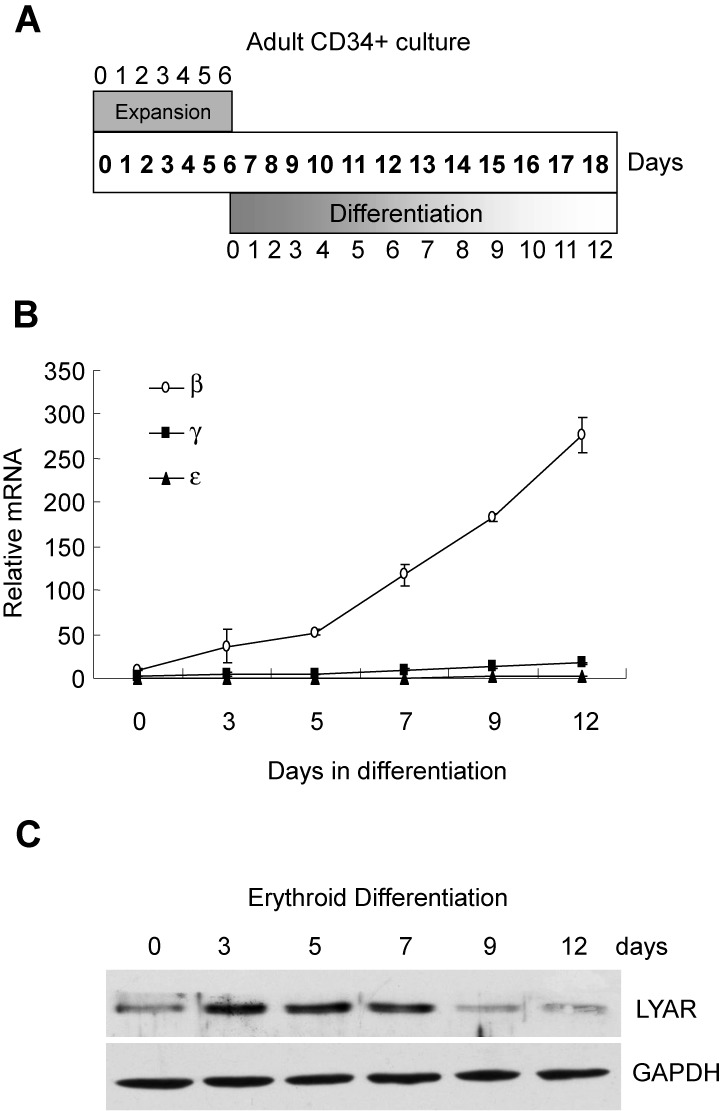
LYAR protein levels and human globin gene expression during erythroid cell differentiation. (**A**) Schematic diagram of *ex vivo* differentiation culture system showing days of expansion and differentiation phases. (**B**) Human ϵ-, γ- and β-globin mRNA levels normalized against GAPDH were detected by quantitative RT-PCR in erythroid cells at indicated days of differentiation. (**C**) Western blot analysis of LYAR and GAPDH from cell lysates of erythroid cells at indicated days of differentiation.

In order to examine LYAR protein levels during human erythroid differentiation, we utilized an *ex vivo* erythroid culture system (20). In this system, purified CD34+ hematopoietic progenitors were first expanded for 6 days followed by differentiation for 12 days in different conditioned media (Figure 5A). As shown in Supplementary Figure S8, this system mimics normal erythroid cell differentiation from undifferentiated blasts at day 0 to orthochromatic normoblasts at day 12 (20,21). By the end of differentiation on day 12, terminal erythroid cells produced great amounts of adult β-globin, low levels of γ-globin and undetectable levels of ϵ-globin (Figure 5B). Of note, γ-globin gene expression was significantly, albeit weakly, induced (Figure 5B). LYAR protein was highly expressed from early (day 3, preproerythroblasts) to mid (day 7, basophilic normoblasts) maturation (Figure 5C). LYAR protein was markedly reduced in more mature (day 9–12) erythroblasts (Figure 5C). The decrease in LYAR protein correlated with the rise of globin mRNA levels during erythroid cell differentiation.

### The role of LYAR on γ-globin expression in human erythroid progenitor cells

**Figure 6.**
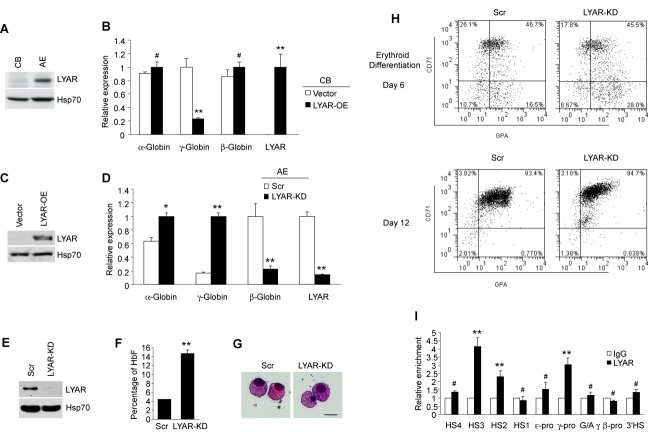
Role of LYAR in developmental globin gene silencing. (**A**) Western blot analysis using indicated antibodies of cell lysate from human erythroid progenitor cells of CB and adult peripheral blood (AE). (**B**) Q-RT-PCR analysis of α-globin, β-globin, γ-globin and LYAR gene expression from CB erythroid progenitor cells overexpressing LYAR (LYAR-OE) or containing vector normalized to β-actin mRNA. Results are shown as mean ± SD from three independent experiments. (**C**) Western blot analysis using indicated antibodies of cell lysate from human erythroid progenitor cells of CB overexpressing LYAR or containing vector. (**D**) Q-RT-PCR analysis of α-globin, γ-globin, β-globin or LYAR gene expression from human AE progenitor cells of LYAR-knockdown (KD) or scrambled control (AE) normalized to β-actin mRNA. Results are shown as mean ± SD from three independent experiments. (**E**) Western blot analysis of LYAR and Hsp70 from cell lysates of Scramble and LYAR-KD erythroid progenitor cells. (**F**) Hemoglobin analysis shows percentage of HbF from Scramble and LYAR-KD erythroid progenitor cells. (**G**) Wright-Giemsa-stained AE progenitor cells at day 12 of differentiation. Scale bar, 50 μM. (**H**) Flow cytometric analysis for CD71 and GPA expression of Scramble and LYAR-KD erythroid progenitor cells on days 6 and 12 of differentiation. (**I**) Localization of LYAR across the β-globin locus measured by ChIP in chromatin fractions from AE progenitors. The precipitated DNA was amplified with primers specific for the indicated regions of the β-globin locus. HS, hypersensitive site; pro, promoter; ^G/A^γ, intergenic region between ^G^γ- and ^A^γ-globin genes. Results are shown as mean ± SD from three independent experiments. ^#^*P*> 0.05, *P< 0.05, **P< 0.01 compared to IgG control.

Human γ-globin gene expression is developmentally regulated, and is gradually silenced after birth. To examine the possibility that LYAR plays a role in this process, western blot analysis was performed using an anti-LYAR antibody in human CB and AE progenitor cells. High levels of LYAR were observed in AE progenitor cells (γ-globin ‘off’ state), whereas barely detectable levels of LYAR were found in CB cells (γ-globin ‘on’ state) (Figure 6A). This is consistent with the putative role of LYAR as a repressor of γ-globin expression.

To examine the effects of altered expression of LYAR in CB and AE progenitor cells, we either overexpressed LYAR protein in CB, or knocked down the levels of LYAR in AE. In CB cells, which have almost no endogenous expression of LYAR, enforced overexpression of LYAR greatly repressed γ-globin expression (Figure 6B and C), whereas α-globin and β-globin gene expression was unaffected by LYAR overexpression (Figure 6B). In AE cells, knockdown of LYAR significantly activated γ-globin expression (Figure 6D and E). We observed a reciprocal high expression of β-globin in Scr cells and low expression in LYAR knockdown AE cells, and a slight increase in α-globin expression (Figure 6D). Hemoglobin analysis indicated that LYAR knockdown led to a substantial increase in HbF compared to the Scramble control (Figure 6F). Neither CD71, nor v-myb was found to increase in LYAR knockdown AE cells (Supplementary Figure S9B). Consistent with this, no morphological differences in cells were observed after LYAR knockdown, suggesting that LYAR may not perturb the overall process of erythroid differentiation (Figure 6G). In fact, flow cytometric analysis of the erythroid surface markers CD71 (transferrin receptor) and GPA demonstrated a similar pattern between Scramble control and LYAR knockdown cells (Figure 6H) at different erythroid differentiation stages.

Next, we examined the distribution of LYAR across the β-globin locus in AE erythroid progenitors using primer pairs spanning key regions of the locus. We found increased LYAR binding on the hypersensitive sites HS3 and HS2 of the locus control region (LCR) and on the γ-globin promoter (Figure 6I). In contrast, we did not observe significant LYAR binding on HS4 or HS1 of the LCR, on the ϵ-globin promoter, on the G/A gamma intergenic region, on the β-globin promoter region or on the 3′HS (Figure 6I). These results imply that LYAR plays an important role in regulating human globin gene expression during developmental processes.

## DISCUSSION

This study was designed to elucidate the role of the nuclear protein human LYAR in γ-globin gene expression. Using immunoprecipitation followed by mass spectrometry analysis, we determined that LYAR interacted with PRMT5, and participated in suppressing γ-globin gene expression through binding directly to the DNA region corresponding to 5′UTR of the γ-globin gene. This was confirmed in both K562 cells and human erythroid progenitor cells.

LYAR was originally isolated from a mouse T-cell leukemia line based on its expression of a Ly-1 epitope in a λgt11 library (29). It is involved in cell growth regulation (29), and has been shown to be important for controlling self-renewal and differentiation of embryonic stem cells (ESCs) (37). In the current study, we observed that LYAR did not affect proliferation of K562 cells (Supplementary Figure S6). This is in line with the notion that LYAR affects growth rate primarily of undifferentiated cells (37). LYAR does not significantly affect growth of ESCs after differentiation has begun (37). We demonstrated that CD71 and v-myb, two erythroid differentiation markers, were not increased after LYAR was knocked down in either K562 cells or human primary erythroid progenitor cells (Supplementary Figure S9). As well, the morphology of human primary erythroid progenitor cells remained unchanged following LYAR knockdown (Figure 6G and H). These results indicate that knockdown of LYAR does not promote differentiation, which is consistent with a role of LYAR in the control of ESC differentiation (37).

LYAR contains a zinc finger and three copies of a nuclear localization signal. At the protein level, human and mouse LYAR share 71% identity and 97% similarity (Supplementary Figure S3). LYAR has eight potential zinc-binding residues (Cys or His) in a cluster (C6HC) at its amino terminus (Supplementary Figure S3), which is similar to the C4HC3 cluster that is frequently associated with chromatin-mediated transcriptional regulation (29,38,39). Thus, LYAR was postulated to have DNA binding capacity (29), but this was never confirmed. In the current study, using CASTing, we identified a nucleic acid binding motif (GGTTAT) to which LYAR binds. We identified a functional LYAR binding motif located in the DNA region corresponding to the 5′UTR of the γ-globin gene (position +26 to +32). Due to its nuclear localization signals, LYAR is localized in the nucleus, predominantly in the nucleolus (29). Using immunofluorescence staining we also demonstrated that LYAR was localized in the nucleus and was almost fully colocalized with PRMT5. That LYAR and PRMT5 functionally interact was indicated by changes to PRMT5-mediated histone marks in the proximal promoter region of the γ-globin gene when the LYAR level was altered.

Human globin gene expression is regulated by transcription factors in a tissue- and developmental stage-specific manner. A number of important transcription factors contribute to this process. A key recent finding is that BCL11A mediates fine tuning of the expression of fetal globins (20). Like other transcription factors, such as KLF1, NF-E4, CP2 and GATA1 (4,40), BCL11A binds the proximal promoters of the γ-globin genes (41) as well as the intergenic region between Aγ-globin and δ-globin, which contains GC-rich motifs (20,21). BCL11A coordinates SOX6, GATA1, FOG1 and NuRD nucleosome remodeling complex to fine-tune human γ-globin gene expression through long-distance interactions within the β-globin locus (21). Previously, we found that NF-E4, a PRMT5-dependent repressor complex, binds in the region of position -53 of the γ-promoter (25,27,28). It has been reported that BCL11A binds to this region as well (41). In the current study, we found that LYAR or a LYAR-containing complex, which interacts with PRMT5, bound the 5′UTR of the γ-globin gene in the region of position +26 to +32. During erythroid *ex vivo* differentiation, LYAR protein exhibited an expression pattern similar to BCL11A and SOX6 (21) (Figure 5C). In EMSA experiments, we identified a high molecular weight band containing LYAR (Figure 3E), very likely representing a LYAR-complex. It will be important to identify other LYAR-interacting proteins and their functions to determine if LYAR coordinates or cooperates with BCL11A or SOX6 to regulate γ-globin gene expression. In addition to BCL11A, other regulatory factors including c-MYB, Ikaros, MBD2, FOP (Friend of PRMT1), NF-Y and ZBP-89 are known to play important roles in globin switching and erythropoiesis (18,42–47).

Several models have been proposed to explain globin switching including gene competition, tracking, chromosomal looping and autonomous gene silencing (1,4). There is experimental evidence to support each model, although the models are not necessarily mutually exclusive. In our studies, when LYAR was knocked down in human AE cells fetal hemoglobin was significantly induced, and β-globin was significantly reduced. This result suggests that there may be a gene competition mechanism involved. In view of the fact that LYAR binds HS3 in the LCR and γ-globin promoter robustly, it is also likely that chromosomal looping facilitates HS sites interacting with preferential genes during development to keep γ-globin genes in the silenced state. In fact, LYAR protein was almost undetectable in CB progenitors but was highly expressed in bone marrow cells. This is in line with the pattern of γ-globin expression during development. However, when LYAR was overexpressed in erythroid cells from CB, β-globin did not exhibit significant reciprocal induction even if γ-globin was markedly reduced. The reason for this is not clear, but is possibly cell context-dependent. Further analysis of the mechanisms of regulation of LYAR gene expression during development or stage-specific patterns will likely provide deeper insight into mechanisms of globin gene switching.

The DNA region corresponding to 5′UTR has been shown to regulate transcription in various species, including humans (48–50). However, the role of the DNA region corresponding to 5′UTR in globin gene regulation remains largely unknown. In the β-globin gene corresponding to 5′UTR, a regulatory region from +10 to +45, the downstream core element, was shown to be important for transcription initiation associated with the transcription factor TFIID (51). Mutations in this element exist in cases of naturally occurring β-thalassemia (52). In the γ-globin gene corresponding to 5′UTR, Stat3 binds at +9 to +16 to repress the expression of the γ-globin gene, and this effect can be reversed by its binding partner, GATA1 (53). In the current study, we demonstrated that LYAR binds to the +26 to +32 region associated with methyltransferase PRMT5. Further studies will determine whether crosstalk between LYAR protein and other transcription factors/regulators on the DNA region corresponding to 5′UTR and LCR modulates β-globin gene expression.

In summary, we demonstrated that LYAR knockdown leads to reactivation of embryonic β-like globin genes in MEL cells and to reactivation of fetal globin in human erythroid cells. These results suggest that the regulation of globin genes by LYAR during mammalian evolution is conserved. Our results indicate that LYAR binds to the DNA region corresponding to 5′UTR of the γ-globin gene, and participates in silencing human fetal globin gene expression, suggesting that LYAR may represent a new therapeutic target for treatment of β-thalassemia and SCD.

## SUPPLEMENTARY DATA

Supplementary Data are available at NAR Online.

SUPPLEMENTARY DATA
