# Supplementary material for: Human fetal globin gene expression is regulated by LYAR
Source: Nucleic Acids Res. 2014 Aug 4;42(15):9740–52. doi: 10.1093/nar/gku718 (PMC4150809; doi:10.1093/nar/gku718)
Supplement: SUPPLEMENTARY DATA [file supp_42_15_9740__index.html]

Human fetal globin gene expression is regulated by LYAR — Human fetal globin gene expression is regulated by LYAR — SUPPLEMENTARY DATA 

# Human fetal globin gene expression is regulated by LYAR

## SUPPLEMENTARY DATA

**Files in this Data Supplement:**

- SUPPLEMENTARY DATA
