# Supplementary material for: Human fetal globin gene expression is regulated by LYAR
Source: Nucleic Acids Res. 2014 Aug 4;42(15):9740–52. doi: 10.1093/nar/gku718 (PMC4150809; doi:10.1093/nar/gku718)
Supplement: SUPPLEMENTARY DATA [file supp_gku718_nar-01796-x-2014-File002.pdf]

SUPPLEMENTARY MATERIALS

Supplementary figure legends

Figure S1. Specificity of anti-LYAR antibody. (**A**) Western blot analysis of cell lysates from K562 cells with pre-immune serum and anti-LYAR antibody. (**B**) Western blot analysis using indicated antibodies of cell lysates from K562 cells stably overexpressing 3HA-LYAR or vector control.

Figure S2. ChIP and Q-RT-PCR primer sequences.

Figure S3. Human LYAR (GI: 49065522) and mouse LYAR (GI: 188219567) protein sequence alignment with Clustal 2.1. A stretch of mostly charged amino acids is boxed. Potential zinc-binding residues in a cluster (C6HC) are indicated by arrows.

Figure S4. Chromatin fractions from K562 cells were immunoprecipitated with anti-LYAR antibody and amplified with primer pairs spanning the indicated regions of the γ-promoter (P-2 to P-1) and γ-gene (P+1 to P+4). Normal rabbit IgG served as the control. $^{\#}P>0.05$, $^{*}P<0.05$, $^{**}P<0.01$ compared to the IgG control.

Figure S5. Total DNA sequence alignment of DNA sequencing results from CASTing. Consensus sequences are boxed.

Figure S6. Cell growth curve of Scrambled control K562 cells (Scr), LYAR

knockdown cells (LYAR-KD1, LYAR-KD2).

Figure S7. LYAR regulates mouse embryonic globin gene expression in MEL cells. (**A**) Western blot analysis with indicated antibodies of lysates from LYAR-knockdown 1 (KD1) and LYAR-knockdown 2 (KD2) or scrambled (Scr) control MEL cells. (**B**) LYAR, εy-globin, βh1-globin, βmaj- and βmin-globin gene expression analyses by Q-RT-PCR of RNA extracted from LYAR-KD1, LYAR-KD2, and scrambled control (Scr) MEL cells normalized to β-actin mRNA. Results are shown as mean ± SD from three independent experiments. **P<0.01 compared to the scrambled control.

Figure S8. Wright-Giemsa-stained adult erythroid progenitor cells at indicated different days of differentiation.

Figure S9. Q-RT-PCR analysis of CD71 and v-myb in Scrambled or LYAR knockdown (KD) K562 cells (**A**) and human adult erythroid (AE) progenitor cells (**B**). Results are shown as mean ± SD from three independent experiments. $^{\#}$P>0.05, *P<0.05 compared to the Scramble control.

Fig. S1

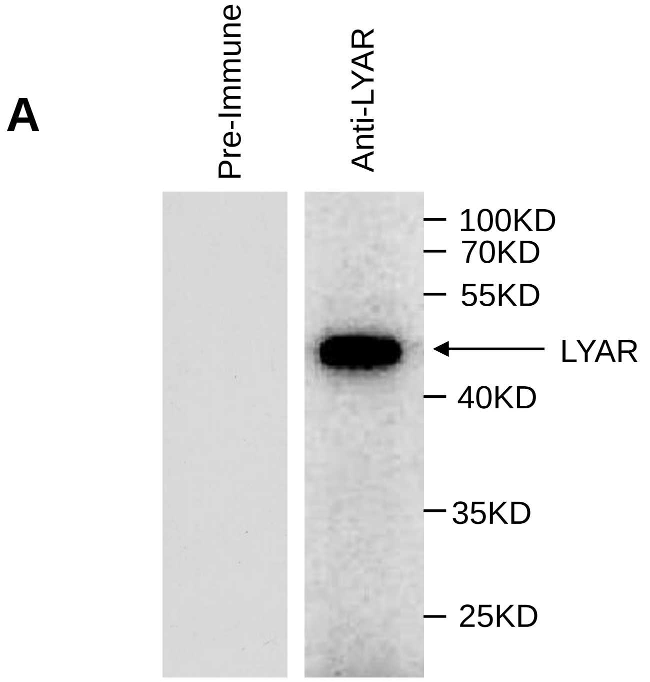
A
Pre-Immune
Anti-LYAR
100KD
70KD
55KD
LYAR
40KD
35KD
25KD

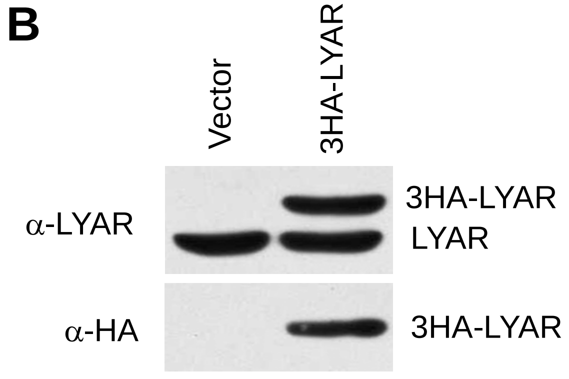
B
Vector
3HA-LYAR
α-LYAR
3HA-LYAR
LYAR
α-HA
3HA-LYAR

Fig. S2

ChIP primer sequences

Locus	5’ Primer	3’ Primer
HS4	AAGGGGTGGACTCCAGAGAC	CTCCTGACTTTCTGTCTAGT
HS3	TATGTATGGGTCAGTGGTCT	CCCTGCTTAGGAGCTTAATC
HS2	AAGCATGAGCAGTTCTGGCCAG	TATGATGCCGTTTGAGGTGGAG
HS1	CATGCAGGACTCTCAAACACTAAC	TCAACAGAGATGGGCAAACCC
ε-pro	TTTTAAGTACCATGGAGAACAGG	ATGAAATGACACCATATCAGATAC
γ-pro	ATCCAGTGAGGCCAGGGGC	GAGATTGACAAGAACAGTTTGAC
G/Aγ	ATCAGCGTGTCATGTCTCAG	CATGAGCTCCTCTAAACCTG
β-pro	TGCTTACCAAGCTGTGATTCC	AACGGCAGACTTCTCCTCAGG
3’-HS	TCACTGAAGTAGGGAGGGAAGAA	AAGGTCATTCCTTTAATGGTCTTTTC
P-1	GAATCGGAACAAGGCAAAGG	GTGGAACTGCTGAAGGGTG
P-2	CAAAAATCCTGGACCTATGC	CCTTTGCCTTGTTCCGATTC
P+1	CACCCTTCAGCAGTTCCAC	CACAAATCCTGAGAAGCGAC
P+2	GTCGCTTCTCAGGATTTGTG	TCACAGTGCAGTTCACTCAG
P+3	TGAGTGAACTGCACTGTGAC	TTTTCAGAGGCACTCCTTAG
P+4	CTAAGGAGTGCCTCTGAAAA	TTCCTCACCCCTGGACATAC

Q-RT-PCR primer sequences

Gene	5’ Primer	3’ Primer
Human		
LYAR	TCCAACAGCGAACCAGTC	ACGGCGTCTTTCACTTTG
α-globin	TGGGGTAAGGTCGGCGCGCA	TGCACCGCAGGGGTGAACTC
β-globin	TCTGTCCACTCCTGATGCTGTTA	ATTAGCCACACCAGCCACCACT
γ-globin	AATGTGGAAGATGCTGGAGGAGAA	CTTCTTGCCATGTGCCTTGACTT
v-myb	GAAAGCGTCACTTGGGGAAAA	TGTTCGATTCGGGAGATAATTGG
CD71	GGCTACTTGGGCTATTGTAAAGG	CAGTTTCTCCGACAACTTTCTCT
Mouse		
LYAR	ATGTGGTGAATCCGTGAAGAAAA	TTGCCTCCGTACTTCTGACCT
εy-globin	TGGCCTGTGGAGTAAGGTCAA	GAAGCAGAGGACAAGTTCCCA
βh1-globin	GAAACCCCCGGATTAGAGCC	GAGCAAAGGTCTCCTTGAGGT
βminor-globin	TCTGCTGTCTCTTGCCTGTG	CCTTTTTGCCATGGGCCTTC
βmajor-globin	GGGTAATGCCAAAGTGAAGGC	GGCCCAGCACAATCACGATCAT

Fig. S3

```
                                ↓   ↓                                 ↓  ↓    ↓    ↓                    ↓    ↓
human           MVFFTCNACGESVKKIQVEKRVSVCRNCECLSCIDCGKDFWGDDYKNHVKCISEDQKYGG 60
mouse           MVFFTCNACGESVKKIQVEKHVSNCRNCECLSCIDCGKDFWGDDYKSHVKCISEGQKYGG 60
                ********************:** **********************.*******.*****

human           KGYEGKTHKGDIKQQAWIQKISELIKRPNVSPKVRELLEQISAFDNVPRKKAKFQNWMKN 120
mouse           KGYEAKTHKGDAKQQAWIQKINELIKKPNVSPKVRELLQQISAFDNVPRKKAKFQNWMKN 120
                ****.****** *********.****:***********:*********************

human           SLKVHNESILDQVWNIFSEASNSEPVNKEQDQRPLHPVANPHAEIS-TKVPASKVKDAVE 179
mouse           SLKVHSDSVLEQVWDIFSEASSSE---QDQQQPPSH-TAKPHAEMPITKVPSAKTNGTTE 176
                *****.:*:*:***:******.**   ::*:* * * .*:****:. ****::*.:.:.*

human           QQGEVKKNKRERKEERQKKRKREKKELKLENHQENSRNQKPKKRKKGQEADLEAGGEEVP 239
mouse           EQTEAKKNKRERKEERQKNRKKEKKELKLENHQENLRGQKPKKRKKNQEAGHEAAGEEAA 236
                :* *.*************:**:************* *.********.***. **.***..

human           EANG-------SAGKRSKKKKQRK----DSASEEEARVGAGKRKR-RHSEVETDSKKKKM 287
mouse           EASGPPEKKKAQGGQASEEGADRNGGPGEDAAEGQTKTAAGKRKRPKHSGAESGYKKKKM 296
                **.*       ..*: *::  :*:    :.*:* :::..****** :** .*:. *****

human           KLPEHPEGGEPEDDEAPAKGKFNWKGTIKAILKQAPDNEITIKKLRKKVLAQYYTVTDEH 347
mouse           KLPEQPEEGEAKDHEAPSKGKFNWKGTIKAVLKQAPDNEISVKKLKKKVIAQYHAVMNDH 356
                ****:** **.:*.***:************:*********::***:***:***::* ::*

human           HRSEEELLVIFNKKISKNPTFKLLKDKVKLVK 379
mouse           HTSEEELLAIFNRKISRNPTFKVLKDRVKLLK 388
                * ****** ***:***:*****:***:***:*
```

Fig. S4

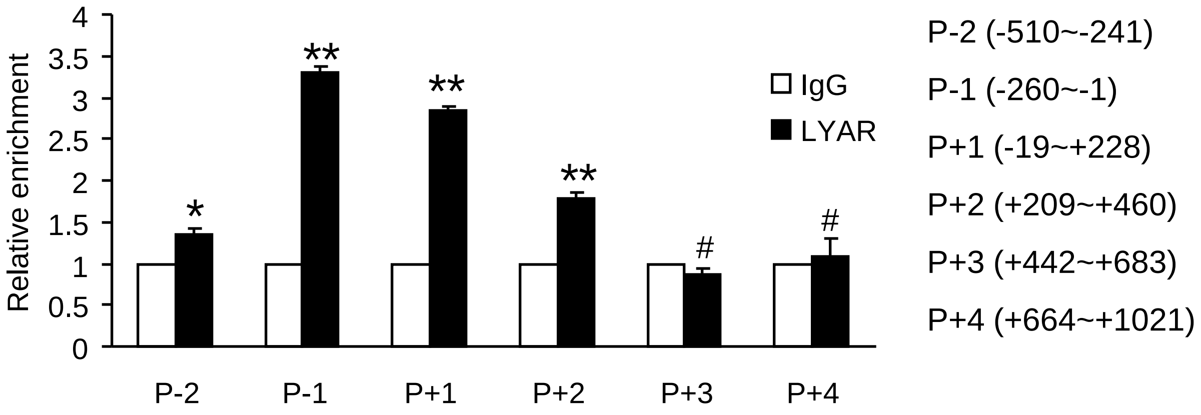

Relative enrichment
4
3.5
3
2.5
2
1.5
1
0.5
0
IgG
LYAR
*
**
**
**
#
#
P-2
P-1
P+1
P+2
P+3
P+4
P-2 (-510~-241)
P-1 (-260~-1)
P+1 (-19~+228)
P+2 (+209~+460)
P+3 (+442~+683)
P+4 (+664~+1021)

Fig. S5

AG**GGTTAT**ATGGGAAAATTAAGG
GTAATT**GGTTAT**GATTGTAGAGTGTT
GTAG**GGTTAT**TGGTACCGATTAGTAG
AACAAGAATCAGGCCTCC**GGTTAG**CG
GAAACCTACGCCGCACTCCA**GGTTAG**
CG**GGTTAG**ATGGTGTGGGCATAGGCT
GAAACCTACGCCGCACTCCA**GGTTAG**
GAGTGTTTGGCCGGTAG**GGTTAG**CTA
CGTGTATAC**CGTTAT**GTGGGCGTAAG
GC**GCTTAT**TGCGGCTCAGGGTGGAAC
G**TGTTAT**CATACTGTCAATTTCTCC
GAAAGGTCGAGT**GGTCAT**CGGGGTCA
CCTACCCTTCCATGGTGCT**GATTAT**A
TATAGCGT**GGTGAT**TAATTTCCATTT
ATGAAG**AGTTAT**AGGGACTGTGAAAT
TGGACAAACAAAT**GGTGAT**GCATTCG
ATCCGGCATTAAGAGA**TGTTAT**GACC
AATATCCTCAATAG**GCTTAT**AGATTG
AAACGGATAACACATTGAA**GCTTAT**C
CCACAGCG**GGTTTA**AATTCAAAGTAC
ATGTTACATTAGGAACCTGTA**GGTTTG**
GTGGGAGA**GGTTTA**GTTTGATGCTTG
ACCGGCAGGTGAG**GATTTT**CGCCTTT
TCTG**CGTTTT**CTGCCTCTGCGGAATG
TTAC**GCTTAG**ACAGTCCTCTTCCGGT
ACTAAGTAGGTGTTGTCG**CGTTGT**AT
CAGTTGTA**GATTGT**TCAGGGGTGGTA
CATACCCTCGCCAGATCA**TCTTAT**GG
GCTCATTGGTCCACGGG**CGTTTT**CT
GTAGGGTTATTGGTACC**GATTAG**TAG
GTAATTGGTTAT**GATTGT**AGAGTGTT
TTGTAACTTCTATGGTGAA**GGTTTC**A
AA**GTTTGT**GATATTTCTGTTCACCTA
G**GGCTCT**AAACTTTATACTATTTGTG
TCTTAATCAGGCAGG**AATTAT**CGGCC
CCCCCAGTGTGGAGG**GGCTGT**TGTAC
TTG**AGTGAT**ATTTTGTCAGTAT
CT**CGTTAG**CGGATGAAACAAAGGGAG
CTCT**GTTTGT**GGCCTGTGAACTATC
TAG**TGTGAT**ATGAAGAGAATAAAAGC
CGAAACGGGTCTCGCGAGAC**AGATAT**
GGGCGCCC**CGTGAT**CTTACCATATAA
AGGGCCTG**GCTTGT**CTTTTGGTGTGT
CGTG**CGTTCT**TTGATATGAGTCCAGG
GCTCTGC**CGTAAT**GAACGATCCCCCG
GTGACCG**GGTTGG**TGTAAGAAGCTCC
GCAG**GGCTAC**ACTTTTCACTTACACG
TAT**GGTTCG**GGTAAGAAACTGGACA
GGTTTCTACTGCAATGGATAAGCCAT
GCACTGCTGTAC**GGGTGT**GCAGACAC
AGCTCCAGCGT**AATTAT**TAATCCCCC
GACCGTCAGTTT**AGTTGT**CTACAGAT
AGTAAATGTGACTTAACGC**GATTAC**A
AACTG**GGTCTT**TCTTATTTATACACT
AGTCTGTGAGC**GATTGT**ACCAGGGTT
GCAG**GGCTAC**ACTTTTCACTTACACG
TGACAAGAGCCGATGCCAA**CATTAT**A
TACTGAT**TGTGAT**GGGGTCAATGTGT
ACGACTAACAAGA**GGACAT**GAAAGGC
CTGGTATGT**AATTAT**TCAACACATGC
ATGTCTAGCGAGAATGGGAT**AGTTGT**
TGAG**CGTTGT**GCCAACGAAGACTTGG
ATGCATGCCAGCACGTCCCA**TATTAT**
CTCG**GGCTCT**TACCGATTAGCTCTTT
ACCTGATGGTAGTCTT**AATTAT**GTTC
ACG**GGCTAA**GTTGCGGTATGAGGGTT
TCACGCATCATACTGA**GGTAGT**TGCC
A**GATTCT**GTGAATGTGCACGGAATGA
AACCCCACCGGTAA**GTTCAT**GAGGCA

Fig. S6

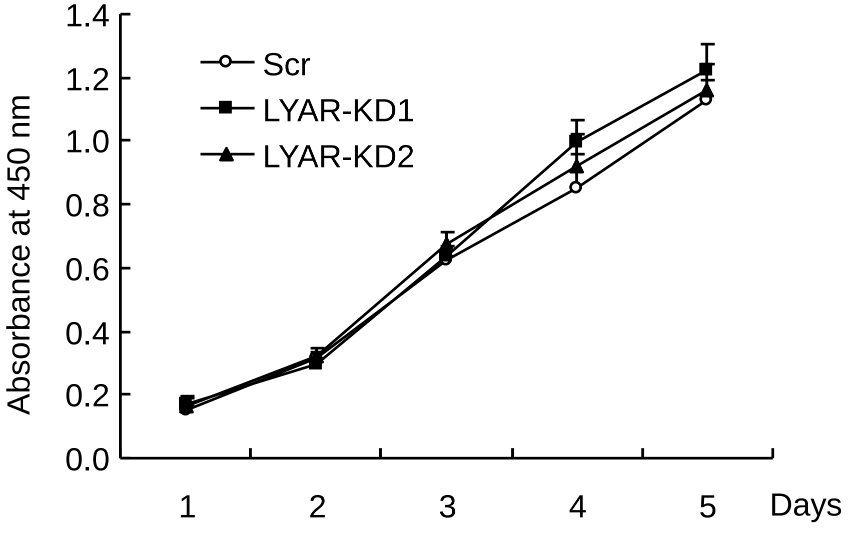

1.4
1.2
1.0
0.8
0.6
0.4
0.2
0.0
Absorbance at 450 nm
Scr
LYAR-KD1
LYAR-KD2
1
2
3
4
5
Days

Fig. S7

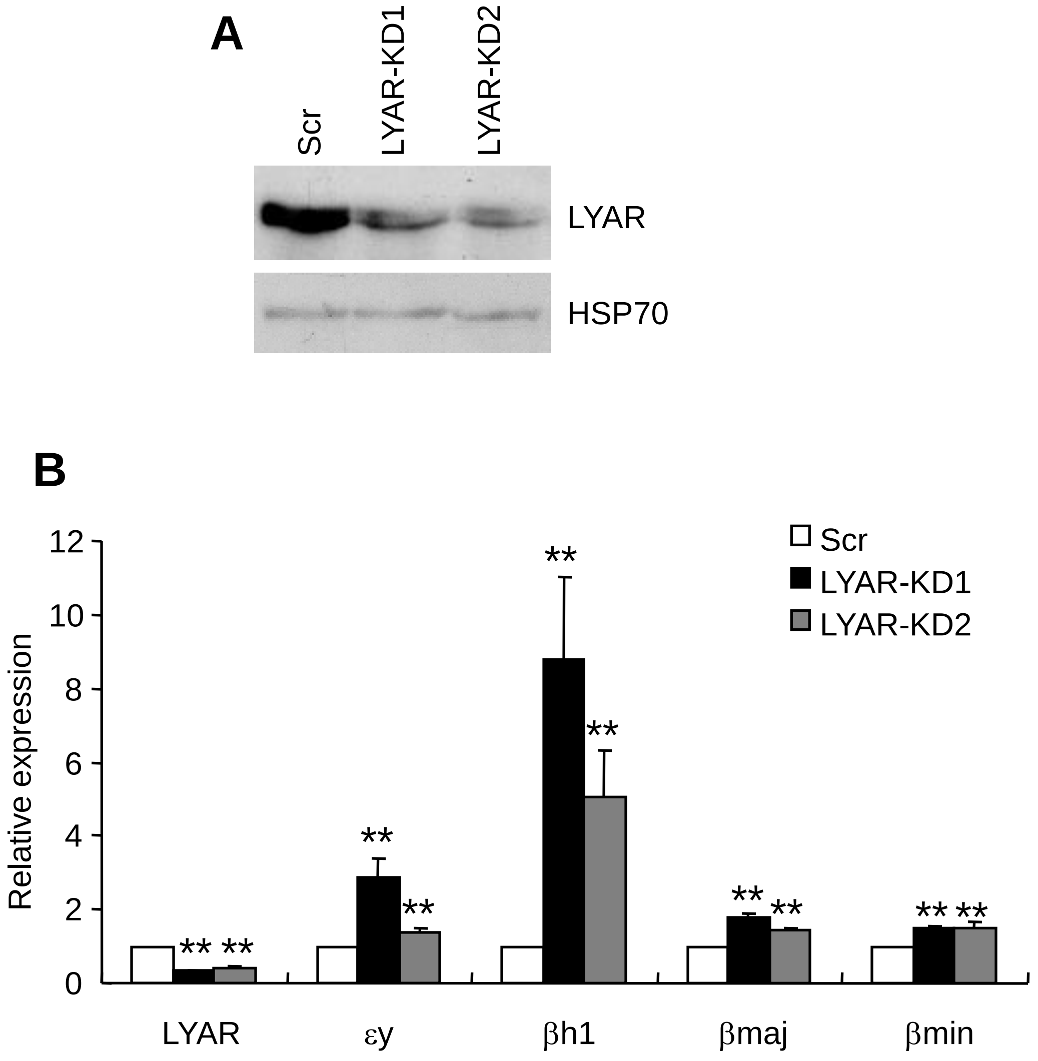
A
Scr
LYAR-KD1
LYAR-KD2
LYAR
HSP70
B
Relative expression
12
10
8
6
4
2
0
**
Scr
LYAR-KD1
LYAR-KD2
LYAR
εy
βh1
βmaj
βmin

Fig. S8

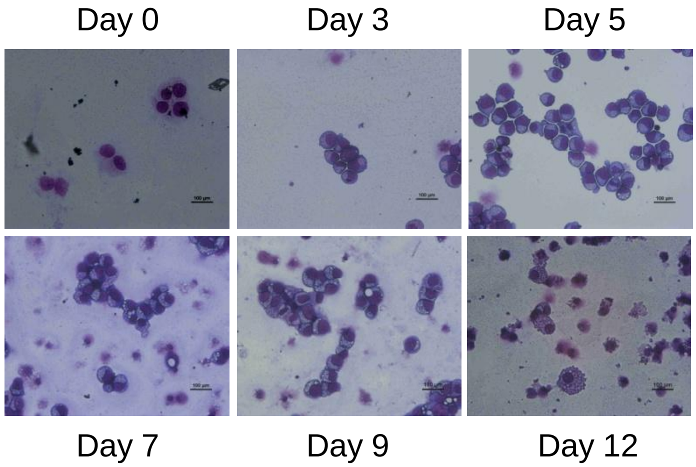

Fig. S9

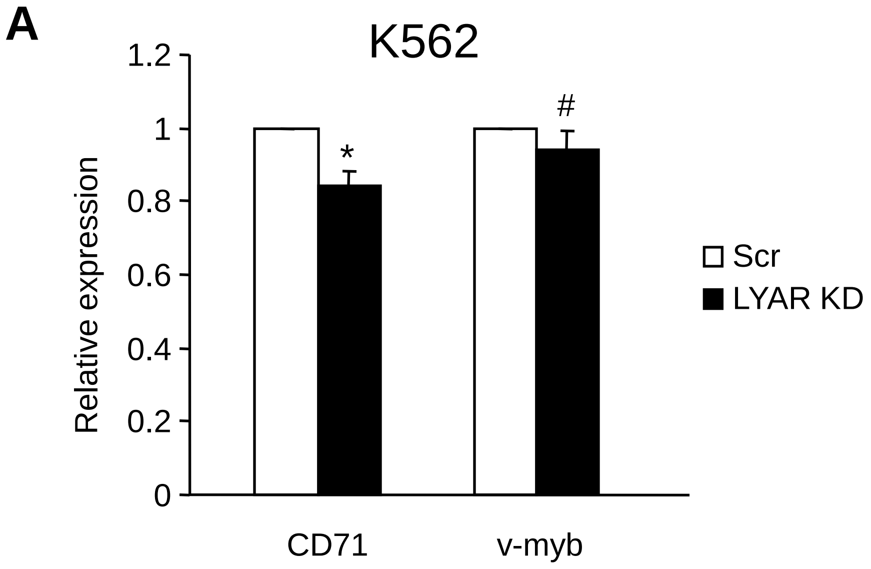
A
K562
Relative expression
1.2
1
0.8
0.6
0.4
0.2
0
*
#
CD71
v-myb
Scr
LYAR KD

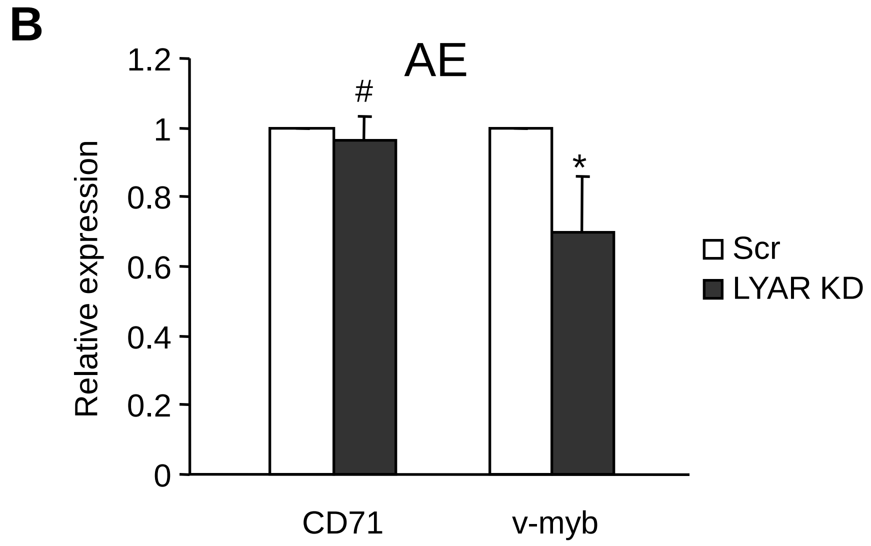
B
AE
Relative expression
1.2
1
0.8
0.6
0.4
0.2
0
#
*
CD71
v-myb
Scr
LYAR KD
